# Acceptability of an on-demand pericoital oral contraceptive pill: a systematic scoping review

**DOI:** 10.1186/s12978-024-01829-7

**Published:** 2024-06-28

**Authors:** Stephen Bell, Susannah Gibbs, Abigail Winskell, Xaviera Villarino, Halle Gill, Kristen Little

**Affiliations:** 1 Independent Research Consultant, Sunshine Coast, QLD Australia; 2 Burnet Institute, Melbourne, Australia; 3 Population Services International, 1120 19 Street NW, Suite 600, Washington, DC 20036 USA; 4 Rollins School of Public Health, Emory University, Atlanta, GA USA

**Keywords:** Scoping review, On-demand contraception, Family planning, Contraceptive preferences, Emergency contraception, Pericoital contraceptive, Reproductive health, Equity, Sexual agency

## Abstract

**Background:**

Access to an on-demand pericoital oral contraceptive pill – used to prevent pregnancy within a defined window around sexual intercourse – could offer women more reproductive agency. A contraceptive with this indication is not currently available in any market. This review aims to understand international user appeal for an on-demand pericoital oral contraceptive pill.

**Methods:**

Systematic scoping review, comprising 30 peer-reviewed papers published between 2014–2023.

**Results:**

Data from 30 papers reporting on research from 16 countries across five World Health Organisation regions suggests widespread user appeal for on-demand oral contraceptive pills that can be used peri- or post-coitally, especially among women who are younger, more educated or who have less frequent sex. Women of varying age, wealth, employment or relationship status, and with different prior experience of using modern contraceptives, were also interested. Women identified clear rationale for use and preference of these types of product: close alignment with women’s sexual lives that comprised unplanned, spontaneous or occasional sex; perceived convenience and effectiveness; discreet use of pills to negotiate contextual circumstances that constrained their reproductive agency. Factors inhibiting use included knowledge barriers and attitudes of service providers, a lack of knowledge and misinformation among end-users, women’s dislike of menstrual side effects and myths related to the effects of hormone content on future fertility.

**Conclusions:**

Introduction of an on-demand pericoital oral contraceptive pill could expand contraceptive choice for diverse women experiencing unmet need for modern contraception and constrained sexual and reproductive agency. Priorities for future research include: broadening the geographical scope of evidence to include SE Asia and the Pacific, and international rural and peri-urban settings; documenting the perspectives of adolescents and unmarried young people; identifying opportunities for innovation in the supply channels to enhance appropriate, affordable access to on-demand oral contraceptives; and unpacking how to bring new pericoital contraceptives to the market in a variety of international settings.

**Supplementary Information:**

The online version contains supplementary material available at 10.1186/s12978-024-01829-7.

## Abstract (plain language summary)

Access to an oral contraceptive pill that is used as needed to prevent pregnancy and taken within a defined window around sexual intercourse (i.e. an on-demand pericoital oral contraceptive pill) could offer women more reproductive agency. Though not currently available in any market, our analysis from this review of international literature reveals widespread appeal among women for using this type of contraceptive product. Clear rationale supporting use and preference included: (1) closer alignment with women’s sexual lives that comprised desired but unplanned, spontaneous or occasional sex than other contraceptives; (2) perceived convenience and effectiveness, offering benefits over other modern contraceptives; and (3) women feeling able to overcome social values and beliefs that constrained their reproductive agency. There were also barriers to use of this type of product, including knowledge gaps and attitudes of service providers, a lack of knowledge and misinformation among end-users, women’s dislike of the side effects, and myths and misconceptions about the impact of the hormone content in pills on future fertility. Introduction of an on-demand pericoital oral contraceptive pill could expand contraceptive choice for diverse women experiencing unmet need for modern contraception and constrained sexual and reproductive agency. Priorities for future research include: broadening the geographical scope of evidence to include SE Asia and the Pacific, and rural and peri-urban settings; documenting the perspectives of adolescents and unmarried young people; identifying opportunities for innovation in the supply channels to enhance appropriate, affordable access to this type of contraceptive; and unpacking how to bring this new contraceptive to the market in a variety of international settings.

**Supplementary Information:**

The online version contains supplementary material available at 10.1186/s12978-024-01829-7.

## Introduction

The rights of all individuals and couples have been at the core of sexual and reproductive health practice and policy since the 1994 International Conference on Population and Development [1] and the 1995 Fourth World Conference on Women [2]. Today, issues of reproductive agency, gender equality and access to modern contraceptives remain firmly established in the United Nations Sustainable Development Goals (UNSDGs) [3] and the Family Planning 2030 (FP2030) commitments [4]. Recent estimates indicate that between 218–270 million women who want to avoid or delay pregnancy are not using safe, modern contraceptive methods [5–7]. Analysis of Demographic and Health Survey data from 52 countries between 2005–2014 illustrated that non-use of modern contraception among married and unmarried women was most often related to current choices not meeting their needs rather than lack of awareness or access to contraception, or high costs [8]. Reasons provided by women included infrequent or no sexual activity, concerns about side effects or health risks associated with contraception, inconvenience of methods, or that they or someone close to them opposed family planning [8]. Contraceptive innovation that brings to market an expanded range of affordable, acceptable, accessible contraceptive products and technologies can enhance women’s and girls’ control over their own contraceptive care if these innovations meet their needs and are reflective of local preferences and lifestyles.

Aligned closely with principles of self-care [9], a women-centred, female-controlled, pericoital on-demand oral contraceptive pill could be used to prevent pregnancy, as needed, within a defined window around sexual intercourse. Though a contraceptive with this indication is not currently available in any market, access to this type of product could offer some women – who want to avoid or delay pregnancy but are not using safe, modern contraceptive methods – more choice, agency and self-determination in reproductive health decision making and action. Contraceptive options that enable discreet, simple use are particularly important in contexts where women’s reproductive agency is constrained in relationships, families and health service settings. One option in the contraceptive development pipeline is pericoital use of a 1.5 mg levonorgestrel (LNG) oral pill as a regular on-demand contraceptive method. Several recent studies have contributed to the body of evidence suggesting that these on-demand pills can be efficacious [10], feasible [11, 12], acceptable [10–12] and safe with limited side effects [10–12]. LNG is a progestin used in many forms of contraception, including emergency contraceptive pills (ECPs). LNG-based ECPs are a form of postcoital pregnancy prevention that are sometimes used proactively and deliberately as an on-demand method immediately after sex [13, 14], in a way that does not align with the typical emergency, back-up use for which ECPs are recommended by health institutions [15]. However, this routine or on-demand use of ECPs is within the scope of the 2015 WHO medical eligibility criteria for contraceptive use [16] which specifies no restrictions on repeated use of LNG ECPs, and the latest WHO self-care guidelines [9] which recommend making over-the-counter ECPs available without a prescription to individuals who wish to use it as an on-demand method.

In 2014, a previous review indicated that demand for an on-demand oral contraceptive pill may be widespread [13]. To update this review [13] and complement findings from recent studies illustrating the efficacy, acceptability, feasibility and safety of pericoital use of LNG [10–12], using a systematic scoping review methodology, the aim of this paper is to understand the current evidence base around potential user appeal for an on-demand pericoital oral contraceptive pill.

## Systematic scoping review methodology

Scoping reviews are a transparent, rigorous and structured method used to synthesise and analyse published literature and identify knowledge and research gaps [17]. This review methodology typically addresses broad research questions to provide an overview and organisation of existing knowledge, rather than a narrow synthesis of a predefined research question [17], and comprises the following stages: identifying a research question or topic; identifying relevant studies; study selection; synthesising and interpreting data; and summarising and reporting on the results [17]. More recently, efforts have been made to improve the systematic nature of scoping reviews in global health, and have been applied to topics such as neglected tropical diseases [18], maternal health [19] and cancer screening [20, 21]. A methodology and guidance for the conduct of systematic scoping reviews has been published [22]; it is this process which we followed in the development of this paper.

Our aim was to undertake a comprehensive review of available published research to explore the user appeal and acceptability of an on-demand pericoital oral contraceptive pill and identify current research gaps and future research priorities. Our two research questions were: What are demographic and behavioural characteristics of actual and potential users of an on-demand oral contraceptive pill? What are the drivers of acceptability and uptake of an on-demand oral contraceptive pill from the perspectives of end users, providers and other influencers?

This review was guided by a review protocol [23], and prepared in accordance with the guidance laid out in the PRISMA Extension for Scoping Reviews [24] (see Additional file 1).

### Definition

Following key elements of a definition used in an earlier review [13], we defined an ‘on-demand pericoital oral contraceptive pill’ as any oral drug preparation that was used in a coitus-dependent manner (i.e. shortly before or after the act of sexual intercourse) to prevent pregnancy. For this review, on-demand use also includes proactive, planned and/or repeat postcoital use of ECPs as a primary contraceptive method (but does not include reactive, back-up use of ECPs up to three days after sexual intercourse, as typically used). Our definition also includes the consumption of other oral drugs perceived as serving a postcoital pregnancy prevention purpose.

### Identification of studies

The following databases were searched on 6th August 2023 to identify relevant papers: PubMed; Web of Science; Global Index Medicus; Scopus. These databases were searched using the following structure of search terms: [on-demand search terms] AND [contraception search terms]. Specific search terms used are detailed in Table 1.

**Table 1:** Search terms

Thematic focus	Search terms	Add with:
On-demand	“on demand" OR on-demand OR pericoital OR peri-coital OR precoital OR pre-coital OR postcoital OR repeat* OR routine OR occasion*	AND
Contraception	Contraception OR contraceptive OR "family planning" OR "Contraception"[Mesh] OR "Contraception, Postcoital"[Mesh] OR "emergency contraception" OR levonorgestrel OR "Levonorgestrel"[Mesh] OR LNG OR postinor* OR "morning after pill" OR "morning after pills" OR "plan b"	AND

To ensure that all relevant papers meeting our eligibility criteria were identified during the search, we also: searched Google Scholar using similar terms and reviewed the first five pages of results; conducted a search of ClinicalTrials.gov and the International Clinical Trials Registry Platform (ICTRP) with simplified search terms; and reviewed the citations from relevant reviews that were uncovered during the literature search.

The results were limited to human studies reporting primary data or original analysis of secondary data published in peer reviewed journals from 2014 to 2023, capturing emerging evidence on use of on-demand pericoital oral contraceptive pills published since previous reviews in 2014 [13, 14]. All populations were considered, inclusive of contraceptive users, potential users, sexual partners, community-based key influencers, health care providers, policy makers and other stakeholders working with a focus on contraception.

Inclusion criteria were not restricted by geographic location, and all publication languages were included. Members of the review team speak English, French and Spanish; DeepL Translator was used to translate titles and abstracts in Portuguese. Papers were excluded if the research was not peer reviewed; did not contain primary data or original analysis of secondary data; and did not align with the definition of an on-demand oral contraceptive pill noted above. Unpublished grey literature, conference abstracts, conference reports and media articles were also excluded.

Records were deduplicated across databases, and title/abstract dual review was conducted in pairs by members of the research team (KL, HG, XV, SG). Kappa statistics for reviewer pairs ranged from 0.22 to 0.43. Reviewers discussed and resolved any discordances and recorded an exclusion reason for each record. We then obtained the full text of all articles identified as potentially relevant during the title/abstract review and proceeded to a combined round of full text review for inclusion and data extraction. SB independently reviewed each paper in the full text review and conferred with KL on results to confirm inclusion of the publication in the review.

### Data extraction and synthesis

The final selection of papers identified for inclusion were reviewed using a data extraction tool designed by the authors for this scoping review. Data extraction fields are outlined in Table 2.

**Table 2:** Data extraction fields

Journal information & study overview	On-demand contraceptive information
- Publication citation - Funding source - Study setting - Population characteristics - End-user vs service provider vs stakeholder perspective reported? - Dates of data collection - Sample size - Analysis technique - Theoretical framework - Ethical considerations	- Study definition of ‘on-demand contraception’ (inc. frequency of use) - Frequency of sexual activity in study population - Demographic and behavioural characteristics of users/potential users (RQ1) - End-user, provider and stakeholder perceptions on acceptability/uptake of on-demand pill (RQ2)

Two types of information were collected. The first included referencing information, study population, location of study, and a description of research methods and analysis procedures. The second extracted findings deductively from each paper in relation to the two research questions. Descriptive quantitative data was extracted primarily to tackle research question 1, while qualitative data was extracted primarily to explore research question 2 (see Table 2). Further inductive synthesis analysis of the qualitative data extracted from each paper followed a thematic analysis approach within each of the research questions following Strauss and Corbin’s [25] system of ‘open’ and ‘axial’ coding. Open coding involves reading through the narrative data to increase familiarity with the material and to prepare ‘theoretical memos’ [25] as analytical reminders for generating ideas and making links between different findings. Axial coding describes the later process of linking or organising open codes into themes and sub-themes, providing evidence to support thematic findings. The following findings section is structured around these research questions and themes.

## Results

**Fig. 1:**
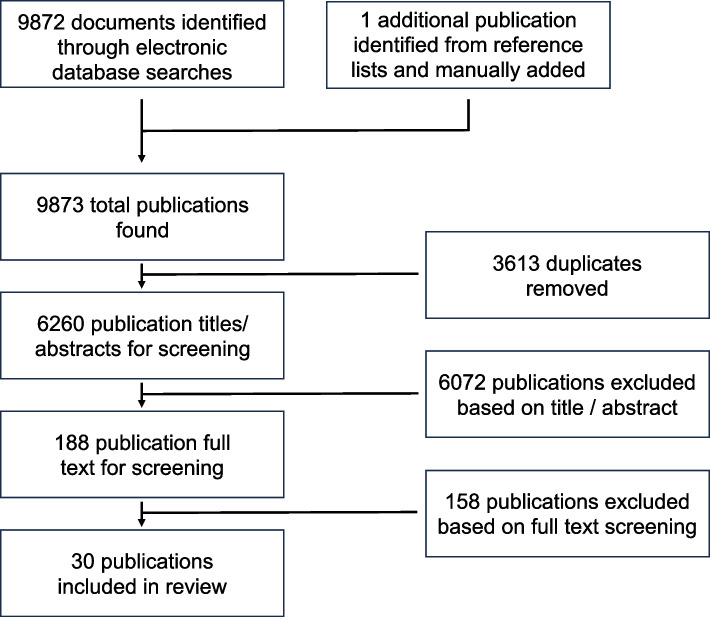
Flow chart showing the selection process

A total of 6260 unique references were identified; after screening, 30 papers met the inclusion criteria for the scoping review (see Fig. 1). The characteristics of the final 30 papers are summarised in Table 3.

**Table 3:** Summary of paper characteristics

Citation	Contraception	Contraceptive use	Study design	Study population and sample size	Location
***International***					
Festin et al. [10]	Pericoital oral pill (LNG 1.5 mg)	Mean monthly pill intake of 4.3–6.2	Trial	Women, 18–45 yrs (*n* = 330)	Urban cities; Thailand, Brazil, Singapore, Hungary
***Africa***					
Both & Samuel [26]	ECP	Repeat ever use: 64% more than once, incl. 34% more than 10 times	Qualitative	Young people, 15–29 yrs (*n* = 66); Pharmacists (*n* = 8); stakeholders: (*n* = 3)	Urban city; Ethiopia
Chin-Quee et al. [27]	Pericoital oral pill (LNG 1.5 mg)	Hypothetical use	Quantitative survey	Women, 18–49 yrs (*n* = 6162)	Urban cities; Kenya, Nigeria
Chin-Quee et al. [28]	ECP	ECP was main contraceptive for 15% in Nairobi and 41% in Lagos	Quantitative survey	Women, 18–49 yrs (*n* = 1022)	Urban cities; Kenya, Nigeria
Fourn et al. [29]	ECP	Repeat ever use: 1–3 times (15%); 4–10 times (4%)	Quantitative survey	Female university students, 16 + yrs (*n* = 570)	Urban city; Benin
Morgan et al. [30]	ECP	Repeat use: 18–48% > monthly; 12–38% main contraception	Quantitative survey	Women, 15–49 yrs (*n* = 12,652)	Urban cities; Kenya, Nigeria
Both [31]	ECP	Repeat use & main contraception	Qualitative	Young people, 18–29 yrs (*n* = 30)	Urban city; Ethiopia
Ajayi et al. [32]	ECP; other postcoital strategy	Self-reported use of ECP as only modern contraceptive	Qualitative	Unmarried female university students, 17–28 yrs (*n* = 56)	Urban towns; Nigeria
Darteh & Doku [33]	ECP	Repeat use: weekly (8%); monthly (25%); occasionally (63%)	Quantitative survey	Male and female university students (*n* = 571)	Urban city, Ghana
Gure et al. [34]	ECP; other postcoital strategy	Hypothetical ECP use	Qualitative	Women, 18–53 yrs (*n* = 21); Pharmacists (*n* = 20); stakeholders: (*n* = 10)	Urban city; Somalia
Hernandez et al. [35]	ECP; other postcoital strategy	Hypothetical ECP use	Qualitative	Women, 15–35 yrs (*n* = 169)	Urban, rural, university settings; DRC
Rokicki & Merten [36]	ECP; other postcoital strategy	Repeat use & main contraception	Qualitative	Unmarried women, 18–24 yrs (*n* = 32)	Urban city; Ghana
Nara et al. [37]	ECP; other postcoital strategy	Hypothetical ECP use	Qualitative	Refugee, 15–49 yrs (*n* = 57); Service providers/stakeholders (*n* = 11)	Urban city; Uganda
Henry et al. [38]	ECP	Ever contraceptive users: 3% only ECP, 3% ECP + traditional; Recent ECP users: 51% used ECP in two months or more in a row	Quantitative survey	Women, 16–44 yrs (*n* = 3703)	Urban city; Ghana
Kalamar et al. [39]	ECP	Repeat use & main contraception	Qualitative	Women, 18–34 yrs (*n* = 299); Men, 18–30 yrs (*n* = 75)	Urban cities; Ghana; Zambia
McCann et al. [11]	Pericoital oral pill (LNG 1.5 mg)	Average peri-coital use 1.72 times/ month; 83% primary contraceptive	Intervention	Women, 18–49 yrs (*n* = 873)	Urban cities, Ghana
Odwe et al. [12]	Pericoital oral pill (LNG 1.5 mg)	Average use of peri-coital pill of 1.3 times per month	Intervention	Women, 18–49 yrs (n = 768)	Urban & peri-urban areas; Kenya
***Americas***					
Brandao et al. [40]	ECP	N/A; provider perspectives	Qualitative	Pharmacists (*n* = 20)	Urban city, Brazil
Provenzano-Castro et al. [41]	ECP	4% used ECP as primary contraceptive	Quantitative survey	Male / female university students (*n* = 1455)	Urban city, Argentina
Biggs et al. [42]	ECP	9/22 participants used ECP as only contraceptive strategy	Qualitative	Women, 15–25 yrs (*n* = 22)	Urban city; US
Barbosa et al. [43]	ECP	Repeat ever use: 2–4 times (48%), 5 + times (20%	Quantitative survey	Women, 15–44 yrs (*n* = 3249)	Urban city, Brazil
Berglas et al. [44]	ECP	9/22 participants used ECP as only contraceptive strategy	Qualitative	Women, 15–25 yrs (*n* = 22)	Urban city; US
Amorim et al. [45]	ECP	N/A; provider perspectives	Quantitative survey	Paediatric physicians working with adolescents (*n* = 151)	Amazonas State, Brazil
***SE Asia***					
Khan et al. [46]	ECP	N/A; provider perspectives	Quantitative, qualitative	Gynaecologists (*n* = 71); GPs & specialists (*n* = 20); stakeholders (*n* = 11)	Urban cities; India
Shakya et al. [47]	ECP	N/A; provider perspectives	Quantitative survey	Community pharmacists (*n* = 227)	Urban districts; Nepal
Panda et al. [48]	ECP	N/A; provider perspectives	Quantitative survey	Doctors (interns, postgraduate trainees, senior resident doctors) (*n* = 200)	Urban hospital; India
Appleton [49]	ECP	Repeat use & main contraception	Qualitative	Women, 20–40 yrs (*n* = 15)	Urban periphery; India
***Eastern Mediterranean***					
Najaji-Sharjabad [50]	ECP	N/A; provider perspectives	Quantitative survey	GPs, midwives & health workers (*n* = 170)	Urban health centres; Iran
***Europe***					
Milosavljevic et al. [51]	ECP	N/A; provider perspectives	Quantitative survey	Gynaecologists (*n* = 166); community pharmacists (*n* = 452)	Serbia
Jambrina et al. [52]	ECP	Repeat ever use: 44% more than once	Surveillance	Women, 16–55 yrs (941 notifications)	Catalonia, Spain
***Western Pacific***					
-	-		-	-	-

Papers reported on research conducted in 16 countries across five WHO regions: nine countries in Africa, including Benin [29], the Democratic Republic of Congo (DRC) [35], Ethiopia [26, 31], Ghana [11, 33, 36, 38, 39], Kenya [12, 27, 28, 30], Nigeria [27, 28, 30, 32], Somalia [34], Uganda [37] and Zambia [39]; three countries in the Americas, including the US [42, 44], Brazil [40, 43, 45] and Argentina [41]; two countries in South East Asia, including India [46, 48, 49] and Nepal [47]; one country in Eastern Mediterranean, in Iran [50]; and two countries in Europe, in Spain [52] and Serbia [51]. One paper reported on data from multiple international settings [10]; no research was identified from the Western Pacific.

Overall, 27 papers reported on data from urban settings [10–12, 26–44, 46–50], one paper on data from rural areas [35], and one on peri-urban areas [12]. Three papers did not specify urban or rural location [45, 51, 52].

The reviewed papers used qualitative (*n* = 12) [26, 31, 32, 34–37, 39, 40, 42, 44, 49], quantitative survey (*n* = 14) [27–30, 33, 38, 41, 43, 45–48, 50, 51], intervention or trial (*n* = 3) [10–12] and surveillance (*n* = 1) [52] approaches. Twenty four papers reported on the perspectives of end users (e.g., younger women, women of reproductive age, university students, male sexual partners) [10–12, 26–39, 41–44, 49, 52], nine papers on service providers (e.g., pharmacists, GPs, clinicians, midwives, community health workers) [26, 34, 40, 45–48, 50, 51], and four on other stakeholders (e.g., donors, policy makers, NGO workers) [26, 34, 37, 46].

With regard to type of on-demand oral contraceptive pill, 26 papers reported on ECPs [26, 28–52] and four on a pericoital pill (1.5 mg LNG) [10–12, 27]. Repeat use of ECPs, or use of ECPs as a primary contraceptive method, by end-users was documented in 16 papers [26, 28–33, 36, 38, 39, 41–44, 49, 52], use of pericoital pills in 3 papers [10–12], and hypothetical use of ECPs or pericoital pills in 4 papers [27, 34, 35, 37]. Seven papers reported provider perspectives of repeat use of ECPs [40, 45–48, 50, 51]. Six papers from the African region reported use of other postcoital prevention strategies – five on non-contraceptive drugs [32, 34–37], and three on other non-medical strategies [32, 34, 38].

### Characteristics of users of pericoital contraceptive pills

Key characteristics of women who reported use of, or hypothetical interest in using, on-demand pericoital pills – in the form of pericoital LNG or postcoital ECPs – are presented in Table 4.

**Table 4:** Characteristics of users of peri- or post-coital pills

Characteristic	Summary findings
Age	13 papers reported on age characteristics [11, 12, 26–32, 36, 38, 43, 44]: - Two papers reported likely use / adoption of a pericoital pill among women aged 18–49 years [11, 12] and one among women aged 18–34 years [27] - Ten papers reported repeat use of ECPs for pregnancy prevention among ‘young people’ [31], students [29, 32], and women in their 20 s [26] or aged 15–25 years [44], 16–24 years [38], 18–24 years [36], 20–24 years [30] or 18–34 years [28, 43] - One paper reported greater likelihood of repeat use of ECPs among a subset of women aged 35‒49 years than women aged 18‒34 [28]
Education	Seven papers reported on education characteristics [12, 27, 28, 30, 31, 38, 43]: - Four papers reported ECP-users as more likely than non-users to have attained higher levels of education [28, 30, 38, 43] - One paper reported women with secondary or post-secondary education were more likely than women with primary education or less to endorse a pericoital pill [27] - Two papers indicated no significant variation by education between adopters and non-adopters of a pericoital pill [12] or ECPs [31]
Frequency of sex	11 papers reported on frequency of sex [10–12, 26–31, 36, 39, 42]: - Nine papers reported use of peri- and postcoital pills among people having infrequent, occasional, or irregular sex [10–12, 26, 27, 29–31, 39] - One paper reported greater likelihood of repeat use of ECPs among women who had sex more frequently (2–3 times a week) as compared to those having sex less frequently (once per week, < once per week) [28] - One paper indicated frequency of sex had no impact on ECP use [30]
Wealth / employment	Five papers reported on socio-economic status [26, 28, 30, 38, 43]: - Three papers reported that ECP users were employed [30], had their own income [43], or had a higher relative income [30, 38] - One paper reported repeat ECP use among lower income women [28] - One paper indicated socio-economic background had no impact on ECP use [26]
Relationship status	11 papers reported on relationship status [11, 12, 26–28, 30, 31, 36, 38, 43, 44]: - Use of ECPs among women who were unmarried / never married [26, 30, 31, 36, 38] or single [28], and women who were married [28] or with a ‘steady’, ‘committed’ or ‘serious’ partner [43, 44] - One paper reported no significant variations between adopters and non-adopters of a pericoital pill by marital status [12] - One paper reported women who were separated, divorced or widowed as less likely than single women to sanction a pericoital pill [27] - One paper reported use of a pericoital pill among women with a ‘committed’ or ‘serious’ partner [11]
Previous use of contraceptives	Eight papers reported on previous use of contraceptives [11, 12, 27, 28, 35, 38, 43]: - Women who had previously discontinued using modern contraceptives / never used a modern method were willing to use a pericoital pill as a primary method [11, 12] - Women who had ever used ECPs or short-acting contraceptives [27], or any modern method [12], were more likely to use a pericoital pill [27] - Women who had used modern contraceptives were less likely to have used ECPs multiple times [28, 35, 38] - Women with previous abortion experience had used ECPs repeatedly [43]

In summary, a greater number of papers reported use of ECPs among younger women aged 15–34 years [26, 28–32, 36, 38, 43, 44] and pericoital pills among women of wider reproductive age range [11, 12, 27]; and pericoital pills and ECPs among women with higher levels of education [12, 27, 28, 30, 38, 43] and among women reporting less frequent sex [10–12, 26, 27, 29–31, 39]. Papers defined ‘infrequent’ as up to 6 times per month [10–12, 30] or less than weekly [30]. As illustrated in Table 4, papers provided disparate conclusions about the use of pericoital pills among women with differing characteristics related to wealth, employment, relationship status or previous use of contraceptives. We were also unable to explore issues associated by urban, peri-urban or rural place of residence due to the lack of data from settings that were not urban.

### Acceptability of on-demand contraceptive pills

#### Alignment with sexual lives and ability to plan

Ten papers – focusing on ECPs in Ethiopia [26], India [49], Ghana [36, 39], Zambia [39], DRC [35] and the US [42, 44], and a pericoital pill in Ghana [11], and Thailand, Brazil, Singapore and Hungary [10] – documented how pericoital contraceptive pills were perceived to align well with sexual lives that comprised desired but unplanned, spontaneous or occasional sex. Participants’ sexual lives included occasional sex at weekends or holidays among young people living with parents [26]; long-distance relationships, or having a partner not always at home [10, 26, 39]; casual sex [36]; or surprise, spontaneous sex with a sexual partner [35]. Reasons given in studies included that ECPs can be taken only when needed [36, 39, 44, 49], and the avoidance of common errors arising with other hormonal methods, such as forgetting a dose or letting a method lapse [10, 35, 39]. A 30-year-old woman in a study in India preferred repeat use of ECPs as she did not see her boyfriend on a regular basis so it made no sense for her to keep ingesting “unnecessary pills” [49]. Amber, aged 15–25 in a study in the US [44], said, “There's no challenges [using ECPs]. That's why I like it. It's not a consistent thing. It's based on my sex life. So, if I don't have a sex life, then I don't have [ECPs]”.

Participants in two of these studies explained that use of pericoital pills enabled them to plan to prevent pregnancy [11, 39], including use of pericoital LNG pills before sex [11] and the possibility of buying multiple doses of ECPs to have when needed [39]. In a study in Ghana and Zambia [39], one man aged 18–30 years said, “unplanned sex is why I was saying you need to buy in bulk, because you don’t know when fire will come. It is always better to prevent than to cure”. A women aged 18–24 years from the same study [39] said:

“Sex just happens, its unpredictable, you could not predict it. So, it is better it is just home or in your handbag wherever you go… And if you have the finance supporting you, then you can buy as many as possible”.

#### Navigating relational power imbalances and conflict

Five papers – focussing on ECPs in Ethiopia [26, 31], Kenya and Nigeria [28], the DRC [35], Ghana [36] and Somalia [34] – reported how women could repeatedly use ECPs to navigate power imbalances or conflict in their relationships. Some women used ECPs to deal with a lack of sexual decision-making power, including where partners refused to use a condom [28]; a lack of trust in men to practice withdrawal [36]; or young women relying on their partners financially for school fees and personal items, and providing sex in return [36]. Another reason was women’s difficulties negotiating condom use with a sexual partner at point of intercourse [35]. A young urban woman aged 15–19 years from a study in the DRC [35] explained, “[ECPs are] easy to use compared to the condom, where you have to ask the boy to put it on. But [ECPs] I can take before or after we have intercourse without the boy knowing anything, and it’s done”.

Secretive repeat use of ECPs also enabled women to deal with differing reproductive intentions within relationships [26, 31, 34]. In a study in Ethiopia [26, 31], Mï’ïraf, a 30-year-old woman and NGO worker with an MA degree, explained how she had used ‘Postpill’ (a locally-branded ECP) at least six times in her current relationship because her partner kept asking her to have a baby with him. She believed his requests were an indirect way of asking her to marry him, but she was not sure about the relationship and a pregnancy would mean staying together.

#### Coping with socially-constrained contexts

Four papers – in Ethiopia [26, 31], India [49], Ghana and Zambia [39] – illustrated how repeat ECP use enabled unmarried women to navigate relational, familial and social contexts that prohibited premarital sex and premarital contraceptive use. ECPs enabled unmarried women to keep their sexual lives secret. In many settings, ECPs were reported to be obtained discreetly as an over-the-counter medication in pharmacies and drug stores. As young people in an Ethiopian study explained, ECPs were consumed within 24 hours and only needed to be taken once, so the box and leaflet could be disposed of immediately within the pharmacy, and the pill strip quickly thereafter [26, 31]. In studies in India and Ethiopia [31, 49], this was perceived as particularly important when unmarried women were still living at home with their parents. Rupali, an educated, professional, single, 30-year-old middle class woman, lived with her parents in a city in northern India [49]. She was sexually active and used ECPs as her main contraceptive method in order to have access to birth control in a situation where she and her partner did not want to use condoms, but she could not keep regular monthly contraceptive pills at her parents’ house [49]:

“It is not like I live on my own. Yes, I have my own room, but I don’t lock it or anything. The *bai* (maid) comes for cleaning, mom is in and out. So if I kept it at home and parents found it, can you imagine the *hungama* (commotion) that would cause in the house?”

This scenario was reported by other sexually active single women who were living at their parents’ home in the same study [49].

In a study in Ethiopia, ECPs also enabled young women to navigate social expectations that they should behave in a shy, reserved manner towards sex, which inhibited their ability to prepare for sex by carrying condoms or seeking other modern contraceptives [31]. Unmarried women could use ECPs strategically to prevent pregnancy, but just after sex to avoid reputational damage with their sexual partner [31]. Dawit, a young man in this Ethiopian study [31], explained:

“I know [Postpill]. I have also used it [weekly]… We would meet and spend the night together but she didn’t want to have sex. But often in the middle of the night it would happen. So the next morning I would go to get a Postpill from the pharmacy.”

As this quote indicates, another advantage of ECPs is that young men could obtain ECPs for their partner to help manage women’s feelings of shame and anticipated risk of reputational damage and stigma associated with having to ask for contraception prior to marriage [26, 31, 39].

#### Convenient and effective

The convenience and ease of pericoital pills was reported in nine papers [10, 12, 26, 27, 29, 31, 35, 36, 39]. These pills were perceived as easy to access [12, 29], simple and easy to take [31, 35, 36, 39], quick to work [39], discreet and private [10, 31], and reduced stress and worry about unwanted pregnancy after unprotected sex [39].

Respondents perceived ECPs to be trustworthy and effective, based either on personal prior use, or word of mouth recommendations from trusted others who had used ECPs successfully [26, 28, 29, 39, 44]. In one study, 76% of respondents in Nairobi and 78% in Lagos felt that ECPs were as effective as regular oral contraceptive pills [28]. Participants in a study in Ghana and Zambia believed that ECPs would prevent pregnancy if taken correctly [39]. Young women aged 15–25 years in a study in the US expressed confidence in the effectiveness of ECPs, even though they knew that these methods were less effective at preventing pregnancy than other higher-efficacy methods [44]. Olivia, a woman aged 15-25 years from San Francisco, said, “I like how a lot of women have used Plan B after having unprotected sex and it works. It's effective. So that's definitely a reason as to why I like it” [44].

#### Preferred to other modern contraceptives

In seven papers, users of pericoital pills preferred these options to other modern contraceptives [10, 27, 28, 31, 35, 36, 39]. ECPs were preferred over daily contraceptive pills because women did not need to remember to take a daily pill [10, 35, 36]. A young urban woman aged 15–19 years in a study in the DRC [35], said,

“The 28-day pill you are condemned to take every day and we are humans, we may forget one day and then the punishment is that we get pregnant. [ECP] is good because you only have to remember to take [the pill] the day you have sex.”

Participants’ preference for ECPs over condoms was documented in four papers [27, 28, 31, 36]. In addition to overcoming the challenges of negotiating condom use [28, 36] and navigating norms around premarital sexuality in premarital relationships [31] noted above, in a study in Ghana [36], women explained that condoms represented distrust, lack of love and commitment, and promiscuity, were less pleasurable, and were perceived as less reliable due to concerns of bursting or tearing. Participants in two papers [27, 31] were not concerned about sexually transmitted infections (STIs) or HIV due to the substitution of condoms for a pericoital oral contraceptive pill [27, 31]. They explained that a pericoital product would not differ from other effective forms of modern contraception that also do not confer protection [27], and the fear of becoming pregnant often outweighed risk of STIs or HIV [31].

Pericoital and postcoital pills were perceived as preferable to other hormonal options on the basis that a one-time only hormonal contraceptive pill felt or would feel like less of a burden with fewer side effects than a continuous hormonal contraceptive pill [27, 31, 35]. In a study in the DRC, participants suggested that they would prefer ECPs because single-dose regimens seemed less likely to create the rumoured side effects of long-acting reversible contraceptives [35]. In a feasibility and acceptability study of pericoital use of 1.5 mg LNG in Ghana with 873 active participants [11], 97% were satisfied and 96% expressed desire to use the method again in the future if available. Approximately 20% experienced at least one side effect (e.g., vaginal bleeding, headache, cramps, nausea), but 95% of side effects were reported as mild or uncomfortable but tolerable.

#### Using other postcoital oral strategies

Six papers reporting research in African settings [32, 34–38] illustrated that the principle of postcoital oral pregnancy prevention was well-established among women, even though medical contraceptive drugs were not always used. Five papers reported use of medical drugs that were not ECPs [32, 34–37], and three on non-medical strategies [32, 34, 38]. While many of these are not effective pregnancy prevention strategies, these do demonstrate potential user appeal for modern pericoital oral contraceptive options.

Non-ECP drugs women reported using included anti-malarial drugs (quinine, tetracycline) [29, 35, 37], Menstrogen (an abortion pill) [32], gynaecosid (a pill for irregular menstrual cycle) [32], antibiotics [32, 35], Cytotec (for preventing stomach ulcers) [32], Andrews liver salt (laxative and antacid for mild stomach complaints) [32], MNB 760 (for diarrhoea) [32], painkillers (Alabukun, paracetamol) [32, 37] and deworming medicines (Décaris, Tanzol) [35]. In the DRC, women explained that these types of drugs include a warning on the label (i.e., “not recommended during pregnancy”) that is interpreted to mean that they will prevent pregnancy postcoitally [35]. In some instances, women reported using complex regimens of these drugs to fulfil their pregnancy prevention needs. In a study in the DRC [35], one urban woman aged 25–35 years explained:

“Décaris has two pills, but you have to take it the day after [unprotected sex] or it will not work. With Tanzol, you have more time, almost a week, but you have to take all the powder from five pills out in the morning and drink it with water and lime, and then do the same thing in the evening. With the quinine, you also have one week, but if you go more than three days then you need to take 20 pills all at once.”

In studies in the DRC [35] and Nigeria [32], some women talked about regimens which also include ECPs. In Nigeria, some respondents thought that a combination of ECPs (e.g. Postinor I, Postinor 2) and other drugs (e.g. antibiotics, menstrogen) worked best [32].

Non-medical strategies – or “folk remedies” [35] – included drinking strong coffee [29], strongly salted water [29, 32, 36], sodas [32, 36], concoctions including alcohol, lime and potash [32], and ‘yoyo bitters’ and other herbal concoctions [32, 35]. Other strategies included manual extraction of the semen through vaginal douching immediately after intercourse [32, 36], or “jumping really hard to make the sperm come out” [35]. In these papers, these strategies were all employed postcoitally, rather than after a pregnancy had been discovered.

### Barriers to provision and uptake of on-demand contraceptive pills

#### Knowledge barriers among service providers

Six papers – in Ethiopia [26], India [46, 48], Iran [50], Brazil [40], and Argentina [41] – reported knowledge barriers about ECPs among service providers (i.e., doctors, gynaecologists, medical students, pharmacists). Issues included confusion about contraindications [41, 46] and how ECPs work [41, 46, 48], and concerns about health risks associated with repeat use of ECPs [26, 40]. Pharmacists and pharmacy clerks in Brazil were concerned about the destructive bodily effects of ‘uncontrolled’ or ‘indiscriminate’ use of emergency contraception due to the higher dosage of hormones in ECPs compared to oral contraceptive pills [40]. Senior gynaecologists in north India expressed concerns regarding the possibility of ectopic pregnancy and infertility, as well as excessive bleeding during menstruation, vomiting or nausea [46]. The need to educate ECP providers was highlighted as a key requirement to enhance women’s access to ECPs in a range of settings [36, 39, 45–47, 50].

#### Service provider attitudes

Service providers in eight papers – in Ethiopia [26], India [46, 48], Iran [50], Brazil [40, 45], Serbia [51] and Nepal [47] – raised moral concerns about users of ECPs. These included increased ‘promiscuity’ and ‘risky’, ‘irresponsible’ sexual behaviours, with greater likelihood of premarital sex at an earlier age and more sexual partners [26, 40, 45–48, 50, 51], neglect of condom use and risk of STIs and HIV [26, 48, 50], and circumstances where ECPs compromise use of, or replace, other contraceptive products which were perceived as more effective [26, 40, 45, 46, 51]. Physicians in studies in Ethiopia [26] and India [46] stressed that ECPs are a back-up, emergency method, and should not be used as a regular contraceptive method. There were strong reservations about availability of ECPs for younger people, and consequent ‘misuse’ of these drugs as routine rather than emergency contraception, in studies in India [46], Brazil [40] and Nepal [47]. Senior physicians in a study in India proposed solutions that included restricting over-the-counter availability of ECPs, and enacting age restrictions to purchasing ECPs for 18–22 year olds [46].

#### Lack of knowledge and misinformation among end-users

Six papers – in Ethiopia [26], the DRC [35], Ghana [36, 39], Uganda [37] and Zambia [39] – reported knowledge barriers and misinformation among end users. With regard to proactive, planned, on-demand postcoital use of ECPs, knowledge gaps raised by women in studies included not knowing how to use ECPs [26, 36], how frequently ECPs can be used [26, 36], and the possible side effects of ECPs and repeat use of ECPs [26, 37, 39].

Women in some studies who reported regular, routine use of ECPs as their primary contraceptive [26, 36] reported a lack of information sources from which to answer these types of questions. For example, young unmarried women in a study in Ethiopia [26] relied on the information leaflet in the box as the only reliable source of information, written in both Amharic and English, but pointed out the inaccessible language and confusing information. Hiwot, a 22-year-old woman from this Ethiopian study [26] who had used ECPs at least eight times, said:

“For the Postpills, I went to different pharmacies and I asked them about the side effects of the Postpill. They all said, ‘well, it is this and that’. They all said different things. Then I read the leaflet and it also talks about the advantages, like that it prevents breast cancer. Then someone else says it actually causes breast cancer. So what to believe?”

Misinformation about ECPs was also reported in contexts where women’s postcoital use of a wide range of non-ECP medicines was documented, including in studies in Ghana [36, 39], Uganda [37] and Zambia [39]. These related to incorrect drugs [36], inaccurate regimens [37], how ECPs prevent pregnancy [39], side effects [39], and a belief that ECPs will become ineffective at preventing pregnancy if taken too many times [36]. Such misconceptions inhibited women from adopting ECPs as a modern, more effective form of postcoital pregnancy prevention.

#### End-user concerns with side effects

Similar to health service providers, concerns about the hormonal content of pericoital pills and ECPs, and associated side effects that might be experienced, were noted by some women in studies in Kenya [27], Nigeria [27], Ghana [36, 39] and Zambia [39]. In a safety analysis of an LNG pericoital pill [11], while satisfaction and future intent to use the study method were very high, when women were asked what they did not like about the study method, the most frequent response was that it changed their menstrual cycle. Similar concerns relating to menstrual changes were raised in relation to ECP use in studies in Ghana, Zambia and Kenya [27, 39]. In a Zambian study, one married woman aged 18–30 years said, “I didn’t want to continue using emergency pill each time I had sex. It’s not advisable you can have side effects, you can have prolonged period” [39]. However, some myths and misconceptions about the negative effect of ECPs on future fertility were raised by women in studies in Ghana and Zambia [36, 39]. An 18-year-old Zambian woman explained, “everything has its disadvantage. I have learnt that taking too much of Postinor-2 [ECP], it will come to a time you can’t give birth” [36].

#### Barriers reported less frequently by end-users

Concern about the risk of contracting HIV or STIs when using ECPs was mentioned by a subsample of participants in Kenya [28]. Religious or socio-cultural proscriptions were cited in a study in Kenya and Nigeria as reasons for not using a hypothetical pericoital pill [27], and religious reasons for not using ECPs in a study in Benin [29]. Prohibitive out-of-pocket costs were mentioned as a barrier to using ECPs by Congolese refugees – specifically women with no income – in Uganda [37]. Women described feelings of anticipated stigma about procuring ECPs from government clinics and hospitals in studies in Ghana and Zambia [36, 39].

## Discussion

Data from 30 papers published since 2014 – reporting on research conducted in 16 international settings across five WHO regions – suggests widespread user appeal for on-demand oral contraceptive pills that can be used within a defined window around sexual intercourse. These include studies documenting use of pericoital LNG pills [10–12], hypothetical support for use of pericoital LNG pills [27, 34, 35, 37], and repeat use of ECPs or routine use of ECPs as a primary contraceptive method [26, 28–33, 36, 38, 39, 41–44, 49, 52].

The results of this scoping review extend the findings from a previous review conducted on this topic in 2014 [13], similarly confirming widespread demand for an on-demand oral contraceptive pill. We extend this review in two ways: by identifying a further 30 papers published since 2014, which report on research from 10 different countries and two different WHO regions; and identifying and categorising clear rationale for women’s use of these products in their daily lives.

Among both women using, or interested in using, on-demand pericoital oral contraceptive pills, our analyses indicate clear appeal among women who are younger (aged 15–34 years), more educated and who have less frequent sex [10–12, 26–32, 36, 38, 39, 43, 44]. There is also broader interest among other women, including women of wider reproductive age range [11, 12, 27], varying wealth or employment status [26, 28, 30, 38, 43], and with diverse marital or relationship status [11, 26, 28, 30, 31, 36, 38, 43, 44]. Reviewed papers noted use or acceptability of pericoital pills among women who had never used a modern method or previously discontinued using modern contraceptives [11, 12], and women who had ever used ECPs or short-acting contraceptives [27]. These findings suggest that offer of an on-demand pericoital oral contraceptive pill could increase overall use of modern contraceptives, including among women who have infrequent sex and among women who find that available methods do not meet their needs.

Study participants in diverse international studies provided clear rationale as to why they used or preferred pericoital oral contraceptive pills. First, pericoital pills aligned more closely with some women’s sexual lives than other contraceptive options [10, 11, 26, 35, 36, 39, 42, 44, 49]. This was particularly the case for unmarried women, women who did not see their sexual partners all the time, women with sexual lives that involved spontaneous or occasional sex, and women who did not want to, or could not, plan for sex using other women-controlled modern strategies without having to be on a long-acting hormonal contraceptive. A new form of on-demand pericoital pill – or repeat use of ECPs as per latest WHO medical eligibility criteria for contraceptive use [16] – enables women to plan deliberately to prevent unintended or unwanted pregnancy, but just doing so after sex has occurred. This is a rather different, more agentic strategic framing of postcoital use of ECPs than is currently assumed under the branding of this medicine as being for emergencies, or as a back-up option.

A second rationale, for some women, related to individual-level choices and preferences. Women perceived on-demand pills as convenient and effective [10, 12, 26, 27, 29, 31, 35, 36, 39], and offered benefits over other modern contraceptive options [10, 27, 28, 31, 35, 36, 39]. Examples included not having to remember to take a daily pill [10, 35, 36]; overcoming the challenges of negotiating condom use [28, 36] or navigating socially-constructed associations between condoms and distrust, lack of love and commitment, and promiscuity [36]; and fewer perceived side effects associated with a one-time only hormonal contraceptive pill compared with continuous hormonal contraceptive options [27, 31, 35].

The seemingly widespread use of other postcoital oral strategies that do not rely on modern hormonal solutions to pregnancy prevention – as described in six papers reporting research in African settings [32, 34–38] – is further evidence of women’s reproductive agency. These findings illustrate that the strategic principles of women-controlled, on-demand postcoital oral pregnancy prevention are well-established among women with unmet need for modern contraception, or lack of access to, experience with, knowledge of, or trust in Western contraceptive products and technologies. An effective pericoital oral contraceptive pill that is made available and accessible to women who do not currently use modern contraceptives, especially for those who use unproven or potentially dangerous non-contraceptive methods to prevent pregnancy, could be an important step toward meeting the UNSDG targets and FP2030 commitments.

Third, on-demand pericoital oral contraceptive pills enabled women to strategically negotiate contextual circumstances that typically constrained their reproductive agency. This type of contraceptive method enabled women to navigate challenging relational contexts where power imbalances and conflict in relationships typically led to less choice and control over becoming pregnant [26, 28, 31, 34–36]. Evidence also indicated how discreet on-demand oral contraceptive pills helped women navigate restrictive social and familial contexts that stigmatize premarital sex and use of modern contraceptives before marriage [26, 31, 39, 49]. In this sense, our analyses illustrate that understanding the potential market for an on-demand oral contraceptive pill is as much about the women who might choose to use it, as it is about understanding the characteristics of the social contexts within which women are able (or not) to enact agency in their sexual and reproductive decision-making and action.

Our analyses also identified a range of barriers to provision and uptake of on-demand pericoital oral contraceptive pills, which were largely associated with ECPs. Key barriers among service providers related to a lack of knowledge [26, 40, 41, 46, 48] and attitudes that framed users of ECPs as a postcoital strategy as immoral, promiscuous, risky and irresponsible [26, 40, 45–48, 50, 51]. Key barriers among end users related to a lack of knowledge, misinformation and a lack of trusted information sources [26, 34–37, 39], a strong dislike of experienced side effects of ECPs on menstrual health [27, 36, 39], and myths and misconceptions linking the negative effects of hormones in ECPs on women’s future fertility [27, 36, 39]. Although predominantly associated with ECPs, these barriers allude to some of the challenges that might be experienced in attempting to bring this type of product to market. However, it could be assumed that a new dedicated on-demand pericoital pill that is branded as a proactive, deliberate pregnancy prevention option – very different to the way ECPs were brought to market as an emergency, back up option, with all the associated social prejudices noted above – with wide ranging health and social value and purpose, supported with simple user information instructions, may not be associated with these barriers to provision and use.

### Study limitations

There are some limitations to this review. We report on 30 papers that documented on-demand pericoital or postcoital use of modern oral contraceptives pills, which is a limited resource base. This is further limited by only four papers reporting on a pericoital pill (1.5 mg LNG) that is [10–12, 27]; 26 papers included in this review report on on-demand proactive, planned and/or repeat postcoital use of ECPs as a primary contraceptive method [26, 28–52]. We have only reported on studies published in peer-reviewed journals and excluded grey literature including government and community reports. Finally, the aim of this review was not to assess the quality of the research involved, but to identify and summarise key themes. However, we have focused on themes that are common across studies as well as ideas reported in fewer papers, to cover both typical experiences and pay attention to the complexity of individual views and experiences across different international settings.

### Future research

Findings identify some clear priorities for future research. First, there is need to broaden the geographical scope of research conducted to date. Over half of the papers under review reported on research conducted in African settings. Only one of the four papers reporting on research conducted in SE Asia provided insight from the perspectives of end-users; yet one-third of married women with unmet need for modern contraceptives in Asia cite infrequent sex as a driver of non-use of contraceptives [8]. Efforts to gather evidence from a diversity of settings across SE Asia and the Pacific would enhance understanding of the potential appeal of on-demand oral contraceptive pericoital pills across these populous but socially, culturally and religiously diverse contexts. Furthermore, the papers overwhelmingly focused on urban settings. A deeper understanding about how on-demand contraceptive pills can contribute to unmet need for modern contraceptive methods among women living in rural and peri-urban areas around the world could increase overall use of modern contraceptives, but also potentially alleviate morbidity and mortality associated with less safe pregnancy prevention strategies currently in use.

Second, research documenting adolescents’ perspectives on on-demand pericoital contraception is scarce. The highest level of unmet need for modern contraceptives is among sexually active 15–19 year olds [5]. Our analyses illustrate how on-demand, woman-controlled contraceptive pills can enable women to navigate the relational, familial, health system and broader social constraints on sexual and reproductive agency in contexts that stigmatize sex before marriage. This type of contraceptive pill could be a potentially exciting solution to helping sexually active adolescents avoid the health and social consequences of unintended pregnancy around the world.

Third, future insight, design and implementation research could identify opportunities for innovation in the supply channels through which women of different ages can access on-demand oral contraceptives in ways that work for them, are closer to home, and alleviate the interference of barriers to uptake. This work applies as much to on-demand pills as it does to other forms of on-demand contraception that are in early-stage development or the future pipeline [53–55].

Finally, future research could unpack how to bring new pericoital contraceptives to the market in a variety of international settings and explore the most efficient regulatory pathways to support the approval of new pericoital products in diverse markets. To support this, we also need to conduct willingness to pay research, with consumers, and explore how to make on-demand pills available (including over the counter, in multi-pill packs, and via different delivery channels) without disrupting sustainable markets for ECPs.

## Conclusion

The current range of contraceptive options has enabled significant sexual and reproductive health progress but is unlikely to help us meet goals outlined in the UNSDGs and FP2030. This paper updates findings from a prior review [13] and complements findings from recent international studies that suggest that these on-demand pills can be efficacious [10], feasible [11, 12], acceptable [10–12] and safe with limited side effects [10–12]. Our analyses – which privilege the socially and culturally located perspectives, preferences, needs and desires of women – indicate that introduction of an on-demand pericoital oral contraceptive pill could expand contraceptive choice for diverse women experiencing unmet need for modern contraception and constrained sexual and reproductive agency across international settings.

### Supplementary Information

Additional file 1.

## Data Availability

No datasets were generated or analysed during the current study.
